# Early influenza vaccine effectiveness estimates using routinely collected data, Alberta, Canada, 2023/24 season

**DOI:** 10.2807/1560-7917.ES.2024.29.2.2300709

**Published:** 2024-01-11

**Authors:** Christa Smolarchuk, Carla Ickert, Nathan Zelyas, Jeffrey C Kwong, Sarah A Buchan

**Affiliations:** 1 Public Health Analytics, Alberta Health, Edmonton, Alberta; 2 Department of Laboratory Medicine and Pathology, University of Alberta, Edmonton, Canada; 3 ICES, Toronto, Canada; 4 Public Health Ontario, Toronto, Canada; 5 Dalla Lana School of Public Health, University of Toronto, Toronto, Canada; 6 Department of Family and Community Medicine, University of Toronto, Toronto, Canada; 7 University Health Network, Toronto, Canada; 8 Centre for Vaccine Preventable Diseases, University of Toronto, ON, Canada; 9 Institute of Health Policy, Management and Evaluation, University of Toronto, Toronto, Canada; * These authors contributed equally to this work and share first authorship

**Keywords:** Epidemiology, Vaccine Effectiveness, Influenza, Influenza Vaccines

## Abstract

Timely and precise influenza vaccine effectiveness (VE) estimates are needed to guide public health messaging and impact vaccine uptake immediately. Using routinely collected laboratory, vaccination and health administrative data from Alberta, Canada, we estimated influenza VE against infection for the 2023/24 season on a near real-time basis, to late December, at 61% (95% CI: 58–64) against influenza A(H1N1), 49% (95% CI: 28–63) against influenza A(H3N2) and 75% (95% CI: 58–85) against influenza B.

Each year, several countries estimate influenza vaccine effectiveness (VE) using the test-negative design. These estimates help to inform how well influenza vaccines are performing [1,2], but are often not timely enough to guide public health messaging during the current influenza season. Employing the use of routinely collected data was critical to understanding real-world effectiveness of COVID-19 vaccines [3-5]. We sought to use routine laboratory and health administrative data, based on a published protocol [6], to provide granular, timely and precise estimates of influenza VE.

## Influenza in Alberta

Alberta, Canada’s fourth most populous province with a population of approximately 4.5 million, provides universal influenza vaccines and healthcare services free of charge. Alberta has province-wide laboratory, health administrative and influenza immunisation databases linkable at the individual level and available on a near real-time basis, which are collected and disclosed based on the Alberta *Health Information Act*. Influenza laboratory testing, influenza vaccination and health administrative data were linked to perform our analysis between epidemiological week 44 (starting 29 October 2023) and week 52 (ending 30 December 2023).

Alberta’s 2023/24 influenza season began in late October with influenza A positivity exceeding 5% during the week of 29 October 2023 (week 44) [7]. At the beginning of October, immunisations were available to residents living in long-term care and on 16 October 2023 (week 42), influenza immunisations were available free of charge to all Alberta residents at pharmacies and public health clinics. By week 46, influenza vaccine coverage for all ages in Alberta was 19.4% and by the end of the study period (week 52), coverage was 23.5% [7]. The dominant circulating strain was influenza A(H1N1)pdm09 (> 90% of cases), but influenza A(H3N2) and influenza B cases were also detected (both < 5% of cases) [7].

## Study population

Cases were people who were PCR-confirmed influenza-positive and controls were people who tested negative for influenza in any setting. To order testing for influenza, a physician must specify on the laboratory requisition that the patient has symptoms of influenza-like illness (ILI); however, there is no standard ILI case definition. According to the Alberta public health laboratory, in the previous season, one third of influenza testing was community-associated, whereas two thirds of testing occurred in a hospital or emergency department. People were considered immunised if they had received an influenza vaccine at least 14 days before the specimen collection date (information on symptoms and symptom onset date was not available). Six people with co-infections were included in the analysis. Those who did not have an Alberta-specific postal code or had gender reported as unknown or non-binary were excluded (< 1% of influenza tests in Alberta). 

## Defining comorbidities

Based on diagnostic codes recorded in past healthcare interactions, individuals diagnosed with at least one of the following were considered to have a comorbidity: asthma, chronic obstructive pulmonary disease, chronic heart, kidney or liver disease, neurological disease (e.g. stroke), diabetes, lupus, Parkinson’s, rheumatoid arthritis, inflammatory bowel disease, chronic substance abuse, HIV infection or solid organ transplantation. Based on pharmacy dispensation records, those dispensed oral corticosteroids (for ≥ 30 days), antineoplastic agents or another immunocompromising drug from a community pharmacist in the 6 months before specimen collection were considered immunocompromised and grouped with those with comorbidities. 

## 2023/24 early vaccine effectiveness estimates

We compared baseline characteristics of cases and controls using standardised mean differences. We used multivariable logistic regression to estimate the adjusted odds ratio comparing the odds of vaccination among cases to the odds among controls. The VE was calculated based on the formula VE = (1 − OR) × 100%. Models were run separately by cumulative week for each influenza type and subtype and all ages combined to assess the stability and reliability of estimates during the initial weeks of the season. We also generated estimates stratified by age group for influenza A(H1N1)pdm09. Models were adjusted for age group (when not stratified by age group), gender, calendar time (week), hospitalisation status and presence of any comorbidity. All analyses were conducted in R Studio Server Pro.

Overall, 38,136 people were included in the analysis, of whom 29,195 tested negative for influenza, 8,325 tested positive for influenza A(H1N1)pdm09, 310 tested positive for influenza A(H3N2), and 312 tested positive for influenza B (Table 1). The oldest age group was more likely to be vaccinated than younger age groups, as were those with a comorbidity. The largest proportion of tests occurred among those aged ≥ 65 years and during the week of 10 December 2023 (week 50).

**Table 1:** Baseline characteristics of the study population analysed in the 2023/24 season vaccine effectiveness estimates, Alberta, Canada, 29 October 2023 (week 44)–30 December 2023 (week 52) (n = 38,136)

Characteristic		Vaccination status					Influenza testing				
		Vaccinated		Unvaccinated		SMD	Test-negative		Test-positive		SMD
		n	%	n	%		n	%	n	%	
Influenza A(H1N1)pdm09											
Overall		9,262	100	28,258	100	NA	29,195	100	8,325	100	NA
Age group	6 months–9 years	544	6	4,321	15	0.31*	3,032	10	1,833	22	0.32*
	10–19 years	99	1	1,496	5	0.24*	1,134	4	461	6	0.08
	20–49 years	1,045	11	8,468	30	0.47*	6,961	24	2,552	31	0.15*
	50–64 years	1,203	13	5,042	18	0.13*	4,718	16	1,527	18	0.06
	≥ 65 years	6,371	69	8,931	32	0.80*	13,350	46	1,952	23	0.48*
Gender^a^	Women	5,093	55	14,764	52	0.05	15,508	53	4,349	52	0.02
	Men	4,169	45	13,494	48		13,687	47	3,976	48	
Comorbidity	No	1,287	14	9,810	35	0.50*	7,637	26	3,460	42	0.33*
	Yes	7,975	86	18,448	65		21,558	74	4,865	58	
Hospitalisation	No	5,245	57	16,650	59	0.05	15,735	54	6,160	74	0.43*
	Yes	4,017	43	11,608	41		13,460	46	2,165	26	
Week	44	395	4	3,059	11	0.25*	3,281	11	173	2	0.37*
	45	596	6	2,654	9	0.11*	2,936	10	314	4	0.25*
	46	807	9	2,862	10	0.05	3,031	10	638	8	0.09
	47	962	10	2,899	10	0.00	2,997	10	864	10	0.00
	48	1,125	12	3,329	12	0.01	3,242	11	1,212	15	0.10*
	49	1,276	14	3,657	13	0.02	3,370	12	1,563	19	0.20*
	50	1,344	15	3,661	13	0.05	3,506	12	1,499	18	0.17*
	51	1,342	14	3,308	12	0.08	3,489	12	1,161	14	0.06
	52	1,415	15	2,829	10	0.16*	3,343	11	901	11	0.02
Influenza A(H3N2)											
Overall		8,287	100	21,218	100	NA	29,195	100	310	100	NA
Age group	6 months–9 years	459	6	2,599	12	0.24*	3,032	10	26	8	0.07
	10–19 years	84	1	1,070	5	0.24*	1,134	4	20	6	0.12*
	20–49 years	911	11	6,184	29	0.47*	6,961	24	134	43	0.42*
	50–64 years	1,031	12	3,726	18	0.14*	4,718	16	39	13	0.10*
	≥ 65 years	5,802	70	7,639	36	0.72*	13,350	46	91	29	0.34*
Gender^a^	Women	4,561	55	11,123	52	0.05	15,508	53	176	57	0.07
	Men	3,726	45	10,095	48		13,687	47	134	43	
Comorbidity	No	1,127	14	6,637	31	0.43*	7,637	26	127	41	0.32*
	Yes	7,160	86	14,581	69		21,558	74	183	59	
Hospitalisation	No	4,571	55	11,393	54	0.03	15,735	54	229	74	0.43*
	Yes	3,716	45	9,825	46		13,460	46	81	26	
Week	44	390	5	2,923	14	0.32*	3,281	11	32	10	0.03
	45	594	7	2,369	11	0.14*	2,936	10	27	9	0.05
	46	756	9	2,297	11	0.06	3,031	10	22	7	0.12*
	47	870	10	2,172	10	0.01	2,997	10	45	15	0.13*
	48	1,004	12	2,279	11	0.04	3,242	11	41	13	0.06
	49	1,094	13	2,330	11	0.07	3,370	12	54	17	0.17*
	50	1,154	14	2,387	11	0.08	3,506	12	35	11	0.02
	51	1,173	14	2,336	11	0.09	3,489	12	20	6	0.19*
	52	1,252	15	2,125	10	0.15*	3,343	11	34	11	0.02
Influenza B											
Overall		8,258	100	21,249	100	NA	29,195	100	312	100	NA
Age group	6 months–9 years	461	6	2,676	13	0.25*	3,032	10	105	34	0.59*
	10–19 years	85	1	1,104	5	0.24*	1,134	4	55	18	0.45*
	20–49 years	907	11	6,183	29	0.46*	6,961	24	129	41	0.38*
	50–64 years	1,025	12	3,700	17	0.14*	4,718	16	7	2	0.50*
	≥ 65 years	5,780	70	7,586	36	0.73*	13,350	46	16	5	1.05*
Gender^a^	Women	4,542	55	11,125	52	0.05	15,508	53	159	51	0.04
	Men	3,716	45	10,124	48		13,687	47	153	49	
Comorbidity	No	1,126	14	6,715	32	0.44*	7,637	26	204	65	0.86*
	Yes	7,132	86	14,534	68		21,558	74	108	35	
Hospitalisation	No	4,552	55	11,449	54	0.02	15,735	54	266	85	0.73*
	Yes	3,706	45	9,800	46		13,460	46	46	15	
Week	44	386	5	2,903	14	0.32*	3,281	11	8	3	0.35*
	45	588	7	2,362	11	0.14*	2,936	10	14	4	0.22*
	46	753	9	2,291	11	0.06	3,031	10	13	4	0.24*
	47	867	10	2,147	10	0.01	2,997	10	17	5	0.18*
	48	1,001	12	2,275	11	0.04	3,242	11	34	11	0.01
	49	1,088	13	2,322	11	0.07	3,370	12	40	13	0.04
	50	1,152	14	2,410	11	0.08	3,506	12	56	18	0.17*
	51	1,171	14	2,378	11	0.09	3,489	12	60	19	0.20*
	52	1,252	15	2,161	10	0.15*	3,343	11	70	22	0.30*

NA: not applicable; SMD: standard mean difference.

^a^ Individuals identifying as non-binary or with unknown gender identification were excluded (< 1% of influenza tests in Alberta).

* Meaningful difference based on SMD > 0.10.

Six people with co-infections are included in this analysis.

The Figure provides weekly cumulative VE estimates from week 44 to week 52 for influenza A(H1N1)pdm09, influenza A(H3N2) and influenza B. An additional breakdown of influenza test result by vaccination status and week is included in Supplementary Table S1. Weekly estimates for influenza A(H1N1)pdm09, Alberta’s current dominant strain (in December 2023), stabilised after week 47, with a shift of only 3 percentage points in the VE estimate from weeks 48 to 52 and a narrowing of the 95% confidence intervals (CI).

**Figure:**
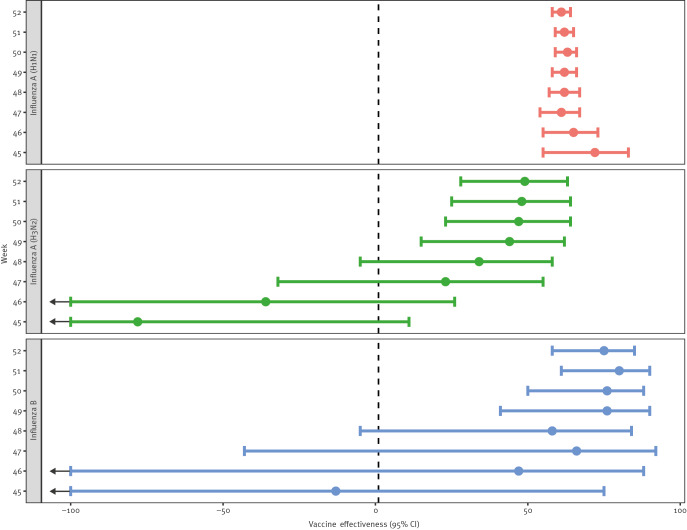
Weekly cumulative influenza vaccine effectiveness estimates by influenza type/subtype, 2023/24 season, Alberta, Canada, 29 October 2023 (week 44)–30 December 2023 (week 52)

CI: confidence interval.

Measures are reported once there were 2 weeks of data. As indicated by black arrows, lower bound 95% CI below −100 for influenza B and A(H3N2) are capped in the figure at −100 to improve readability. Influenza B lower 95% CI for weeks 45 and 46 were −424 and −131, respectively. Influenza A(H3N2) lower 95% CI for weeks 45 and 46 were −264 and −155, respectively.

For the full study period (weeks 44–52), VE was estimated to be 61% (95% CI: 58–64) against influenza A(H1N1)pdm09, 49% (95% CI: 28–63) against influenza A(H3N2) and 75% (95% CI: 58–85) against influenza B (Table 2). The VE against influenza A(H1N1)pdm09 was higher for younger children aged 6 months to 9 years (74%; 95% CI: 66–79) than for adults aged  ≥ 65 years (57%; 95% CI: 52–61).

**Table 2:** Vaccine effectiveness estimates against influenza A(H1N1)pdm09 for all ages and by age group, and against influenza A(H3N2) and influenza B for all ages, Alberta, Canada, 29 October 2023 (week 44)–30 December 2023 (week 52) (n = 38,136)

Age	VE (%)^a,b^	95% CI	Unadjusted VE (%)^b^	Unadjusted 95% CI	Influenza-positive (n)	Influenza-positive vaccinated (row %)	Influenza-negative (n)	Influenza-negative vaccinated (row %)
Influenza A(H1N1)								
All ages	61	58–64	65	62–67	8,325	12	29,195	28
6 months–9 years	74	66–79	72	64–78	1,833	5	3,032	15
10–19 years	62	32–78	58	26-76	461	3	1,134	7
20–64 years	62	57–67	56	51–61	4,079	8	11,679	16
≥ 65 years	57	52–61	43	36–48	1,952	30	13,350	43
Influenza A(H3N2)								
All ages	49	28–63	57	41–69	310	15	29,195	28
Influenza B								
All ages	75	58–85	86	77–92	312	5	29,195	28

CI: confidence interval; VE: vaccine effectiveness.

^a^ Adjusted for age group (when not stratified by age group), gender, calendar time (week), hospitalisation status and presence of any comorbidity.

^b^ Odds ratios were calculated by comparing the odds of vaccination among cases to the odds among controls using logistic regression. Vaccine effectiveness was calculated as VE = (1 − OR) × 100%.

## Validation of estimates using routinely collected data

Generally, VE against medically attended influenza in the community is estimated using data from prospective sentinel surveillance platforms. Since using routinely collected data to estimate VE can introduce biases, such as the inclusion of people who were tested asymptomatically and who are more likely to be tested in hospital, it was important to validate these estimates. Previous work carried out by Kwong et al. assessed biases that may occur when using routinely collected data to estimate influenza VE and found that estimates were valid and comparable to published literature [8]. Canada’s Sentinel Practitioner Surveillance Network (SPSN) has reported Canadian estimates for over 19 years [9], and Alberta, Canada is an SPSN partner through the University of Calgary’s TARRANT Viral Watch (sentinel surveillance network). We generated estimates for three previous seasons and compared them to those reported by SPSN, which systematically swabs patients in the community with ILI on a prospective basis (Table 3). Alberta’s VE estimates were often within 5 percentage points of the SPSN estimates and the CIs were generally more narrow. For example, Alberta’s VE estimate against influenza A(H3N2) for 2022/23 was 52% (95% CI: 48–56), similar to SPSN’s estimate of 54% (95% CI: 38–66), and Alberta’s 2021/22 VE estimate against influenza A(H3N2) was 28% (95% CI: 18–37), comparable to SPSN’s estimate of 36% (95% CI: −38–71) [10,11]. There were no estimates for 2020/21 because there were no seasonal influenza detections in Alberta. Estimates for 2019/20 were comparable to the SPSN interim estimates for most influenza types and subtypes [12].

**Table 3:** Comparison of influenza vaccine effectiveness with estimates from the Canadian Sentinel Practitioner Surveillance Network, by season, Alberta, Canada, 2019/20–2022/23

Season	Influenza type/subtype	Alberta	Canadian SPSN
		VE (95% CI)	
2022/23	Influenza A(H3N2)	52% (48 to 56)	54% (38 to 66)
	Influenza A(H1N1)pdm09	49% (37 to 58)	Not reported
	Influenza B	89% (16 to 99)	Not reported
2021/22	Influenza (any type/subtype)	31% (24 to 39)	36% (−38 to 71)
	Influenza A(H3N2)	28% (18 to 37)	36% (−38 to 71)
2020/21	ND	ND	
2019/20	Influenza (any type/subtype)	48% (45 to 52)	53% (45 to 60)^a^
	Influenza A	39% (34 to 44)	44% (32 to 54)^a^
	Influenza A(H3N2)	32% (22 to 41)	50% (26 to 66)^a^
	Influenza A(H1N1)pdm09	44% (36 to 50)	43% (30 to 54)^a^
	Influenza B	63% (59 to 67)	65% (56 to 73)^a^

CI: confidence interval; ND: No data; SPSN: Sentinel Practitioner Surveillance Network; VE: vaccine effectiveness.

^a^ Interim estimates.

## Discussion

We reported early VE estimates against laboratory-confirmed influenza A(H1N1)pdm09, which stabilised with narrow CIs within 3 weeks of the start of the 2023/24 season. Based on data until the end of December 2023, influenza A(H1N1)pdm09 was the dominant strain in Alberta for the 2023/24 season. The VE against influenza A(H1N1)pdm09 was higher for children than for older adults. Unfortunately, influenza vaccine coverage in Alberta was low among these young age groups (< 20%) [7]. Increasing vaccine uptake among young children in particular may help to reduce the burden of influenza. Importantly, early estimates for influenza B support public health messaging in jurisdictions where influenza B has not yet circulated widely but may increase in 2024.

We were able to provide estimates for influenza B and influenza A(H3N2), which were circulating at low levels, although these estimates were less stable, with wider CIs. The point estimates with wider CIs may shift as more data become available and the CIs may narrow, so timing of reporting VE estimates could pose challenges to public communication and decisions on how early to report estimates. Based on these results, estimates for the dominant strain will probably be available quicker, and with narrower CIs, and later for the non-dominant strains. The VE estimates for dominant strains could be disseminated in public health communications early in the season to support vaccine uptake, with subsequent VE estimates for non-dominant strains disseminated later in the season as estimates improve or as needed in the event of a late season second wave caused by a previously non-dominant strain.

There are a number of limitations using routinely collected health data for this type of analysis. For example, symptom status was not available, and although samples with ILI are indicated when a test is ordered, there is no guidance for physicians on who should be tested for influenza in Alberta. Since we used specimen collected date as a proxy for symptom onset date, the majority of people probably had an earlier onset date, which may have misclassified people as influenza-negative (e.g. a large interval between onset date and testing) or immunised (e.g. a close vaccination date and symptom onset date but a larger interval until testing). In the future, it would be beneficial to conduct whole genome sequencing of influenza viruses in Alberta. This would provide a better understanding the specific viruses circulating and allow us to stratify VE estimates by clade/subclade similar to variant-specific COVID-19 VE estimates [3,5] and analyses conducted by the SPSN [11]. One strength of this design was the ability to use an immunisation registry rather than relying on self-reported immunisation status. Overall, although there are a number of limitations, validation against the SPSN results and the publication by Kwong et al. suggest that these did not have a meaningful impact on the estimates [8]. 

## Conclusion

This work provides a complementary method to estimate VE in a timely manner and for population subgroups which may be difficult to measure using traditional sentinel surveillance platforms. The VE estimates generated from routinely collected data are a useful public health tool that complement sentinel surveillance networks. These methods will be beneficial for timely estimates to guide public health messaging and can impact immunisation uptake in future influenza seasons.

## Supplementary Data

114000 Supplement
