# Supplementary material for: Early influenza vaccine effectiveness estimates using routinely collected data, Alberta, Canada, 2023/24 season
Source: Euro Surveill. 2024 Jan 11;29(2):2300709. doi: 10.2807/1560-7917.ES.2024.29.2.2300709 (PMC10785209; doi:10.2807/1560-7917.ES.2024.29.2.2300709)
Supplement: Supplement [file 23-00709_SMOLARCHUK_Supplement.pdf]

This supplementary material is hosted by *Eurosurveillance* as supporting information alongside the article *Early influenza vaccine effectiveness estimates using routinely collected data, Alberta, Canada, 2023/24 season*, on behalf of the authors, who remain responsible for the accuracy and appropriateness of the content. The same standards for ethics, copyright, attributions and permissions as for the article apply. Supplements are not edited by *Eurosurveillance* and the journal is not responsible for the maintenance of any links or email addresses provided therein.

Supplementary Table 1. Weekly sample by subtype, vaccination status and testing outcome

Week	Vaccination Status	Test Result	Subtype		
			Influenza A(H1N1)	Influenza A(H3N2)	Influenza B
			n	n	n
44	Unvaccinated	Test Negative	2,896	2,896	2,896
	Unvaccinated	Test Positive	163	27	7
	Vaccinated	Test Negative	385	385	385
	Vaccinated	Test Positive	10	5	<5
45	Unvaccinated	Test Negative	5,245	5,245	5,245
	Unvaccinated	Test Positive	468	47	20
	Vaccinated	Test Negative	972	972	972
	Vaccinated	Test Positive	19	12	<5
46	Unvaccinated	Test Negative	7,523	7,523	7,523
	Unvaccinated	Test Positive	1,052	66	33
	Vaccinated	Test Negative	1,725	1,725	1,725
	Vaccinated	Test Positive	73	15	<5
47	Unvaccinated	Test Negative	9,653	9,653	9,653
	Unvaccinated	Test Positive	1,821	108	50
	Vaccinated	Test Negative	2,592	2,592	2,592
	Vaccinated	Test Positive	168	18	<5
48	Unvaccinated	Test Negative	11,897	11,897	11,897
	Unvaccinated	Test Positive	2,906	143	81
	Vaccinated	Test Negative	3,590	3,590	3,590
	Vaccinated	Test Positive	295	24	5
49	Unvaccinated	Test Negative	14,179	14,179	14,179
	Unvaccinated	Test Positive	4,281	191	121
	Vaccinated	Test Negative	4,678	4,678	4,678
	Vaccinated	Test Positive	483	30	5
50	Unvaccinated	Test Negative	16,536	16,536	16,536
	Unvaccinated	Test Positive	5,585	221	174
	Vaccinated	Test Negative	5,827	5,827	5,827
	Vaccinated	Test Positive	678	35	8
51	Unvaccinated	Test Negative	18,855	18,855	18,855

	Unvaccinated	Test Positive	6,574	238	233
	Vaccinated	Test Negative	6,997	6,997	6,997
	Vaccinated	Test Positive	850	38	9
52	Unvaccinated	Test Negative	20,953	20,953	20,953
	Unvaccinated	Test Positive	7,305	265	296
	Vaccinated	Test Negative	8,242	8,242	8,242
	Vaccinated	Test Positive	1,020	45	16
